# Dixon on the Eye, and Althaus on Electricity

**Published:** 1860-05

**Authors:** 


DIXON ON THE EYE, AND ALTHAUS ON ELECTRICITY.—These two works, noticed in the review department of the January and March issues of our journal, have been republished, in superior style, by Lindsay & Blakiston, of this city. They both deserve a place in the library of every intelligent physician.

